# RNA-seq based transcriptomic map reveals new insights into mouse salivary gland development and maturation

**DOI:** 10.1186/s12864-016-3228-7

**Published:** 2016-11-16

**Authors:** Christian Gluck, Sangwon Min, Akinsola Oyelakin, Kirsten Smalley, Satrajit Sinha, Rose-Anne Romano

**Affiliations:** 1 Department of Biochemistry, Jacobs School of Medicine and Biomedical Sciences, State University of New York at Buffalo, Buffalo, NY 14203 USA; 2 Department of Oral Biology, School of Dental Medicine, State University of New York at Buffalo, 3435 Main Street, Buffalo, NY 14214 USA

**Keywords:** Salivary gland, RNA-sequencing, Gene signature, Development

## Abstract

**Background:**

Mouse models have served a valuable role in deciphering various facets of Salivary Gland (SG) biology, from normal developmental programs to diseased states. To facilitate such studies, gene expression profiling maps have been generated for various stages of SG organogenesis. However these prior studies fall short of capturing the transcriptional complexity due to the limited scope of gene-centric microarray-based technology. Compared to microarray, RNA-sequencing (RNA-seq) offers unbiased detection of novel transcripts, broader dynamic range and high specificity and sensitivity for detection of genes, transcripts, and differential gene expression. Although RNA-seq data, particularly under the auspices of the ENCODE project, have covered a large number of biological specimens, studies on the SG have been lacking.

**Results:**

To better appreciate the wide spectrum of gene expression profiles, we isolated RNA from mouse submandibular salivary glands at different embryonic and adult stages. In parallel, we processed RNA-seq data for 24 organs and tissues obtained from the mouse ENCODE consortium and calculated the average gene expression values. To identify molecular players and pathways likely to be relevant for SG biology, we performed functional gene enrichment analysis, network construction and hierarchal clustering of the RNA-seq datasets obtained from different stages of SG development and maturation, and other mouse organs and tissues. Our bioinformatics-based data analysis not only reaffirmed known modulators of SG morphogenesis but revealed novel transcription factors and signaling pathways unique to mouse SG biology and function. Finally we demonstrated that the unique SG gene signature obtained from our mouse studies is also well conserved and can demarcate features of the human SG transcriptome that is different from other tissues.

**Conclusions:**

Our RNA-seq based Atlas has revealed a high-resolution cartographic view of the dynamic transcriptomic landscape of the mouse SG at various stages. These RNA-seq datasets will complement pre-existing microarray based datasets, including the Salivary Gland Molecular Anatomy Project by offering a broader systems-biology based perspective rather than the classical gene-centric view. Ultimately such resources will be valuable in providing a useful toolkit to better understand how the diverse cell population of the SG are organized and controlled during development and differentiation.

**Electronic supplementary material:**

The online version of this article (doi:10.1186/s12864-016-3228-7) contains supplementary material, which is available to authorized users.

## Background

Salivary gland (SG) morphogenesis requires the complex coordination of cells to orchestrate a number of dynamic cellular processes including cell specification, lineage commitment, cell migration, proliferation and differentiation, all culminating in the formation of this specialized gland [1–3]. A network of signaling and regulatory molecules coordinates these vital biological processes, which are accompanied by the dynamic changes in gene expression throughout development. In the mouse, submandibular salivary gland (SMG) morphogenesis occurs over several distinct developmental stages commencing at ~ embryonic day 11 (E11). At this early Prebud stage, the primordial SG fate is established with the thickening of the adjoining oral epithelium. At the next Bud stage, which occurs at approximately E12.5, the thickened epithelium invaginates into the underlying mesenchyme resulting in the formation of a primary bud, which serves as the precursor of the main duct of the salivary gland once the gland reaches maturation. As the embryo develops to E14, the gland undergoes rapid proliferation and intricate branching morphogenesis, during which the end buds undergo successive rounds of clefting resulting in the generation of multiple epithelial buds. This Pseudoglandular stage also coincides with reorganization of the end buds and the formation of the acini, which are the main secretory units of the salivary gland. At the Canalicular stage (E16), the gland is highly branched with lumenization of the main secretory duct nearing completion. The onset of gland cyto-differentiation also occurs at this stage – a process that continues to birth. In the final step of embryonic salivary gland morphogenesis at the Terminal Bud stage (E18), expansion of acini and lumenization of both ducts and acini is almost finished, resulting in a continuous network of ducts connecting the acini to the oral cavity [1, 3–6]. While the gland is functional and able to secrete saliva at birth, further acinar maturation and differentiation continues postnatally, and by puberty, differentiation of the granular convoluted tubules is complete [1, 7].

For many years now, mouse genetic models have been widely utilized to study various facets of salivary gland biology including branching morphogenesis, cleft formation, organ development and differentiation. While these studies have been instrumental in identifying some of the individual genes and signaling pathways necessary for proper salivary gland function, a limited number of studies have focused on the global examination of salivary gland gene expression in mouse [6, 8–10]. In an early study, Hoffman et al. utilized microarrays to examine gene expression profiles at five different stages of mouse submandibular salivary gland embryonic development [6]. In a more recent and complementary study, Musselmann et al. generated a microanatomical atlas of gene expression of embryonic salivary gland by performing laser capture microdissection of distinct epithelial populations obtained from E12.5-E15 mouse embryos [8]. These broad based approaches of global examination of gene expression profiles have been instrumental to deciphering molecular mechanisms of salivary gland morphogenesis and importantly in the discovery of novel signaling pathways such as Fibroblast Growth Factor (FGF) and Bone Morphogenetic Protein (BMP) [6] as well as key signaling molecules such as GSK3β [8] that play important roles in regulating branching morphogenesis in embryonic salivary gland. It is important to note that whereas gene profiling experiments during embryonic development of salivary gland has received much attention, similar in depth studies in adult has been lacking although one recent work has shed some light on aging associated SG gene signature [9].

While microarray technology have been the application of choice in the past for transcriptome analysis, recent advancements have seen the supplanting of microarrays by genomic methods driven by next-generation sequencing (NGS) approaches like RNA-sequencing (RNA-seq) [11]. Compared to microarray technology, deep sequencing based methods provide a more sensitive and precise analytical approach that can accurately quantify gene transcript levels and their isoforms across a broad dynamic range [12]. Indeed, direct comparisons to RNA-seq has revealed the shortcomings of hybridization-based microarray studies such as background noise and saturation and probe set issues such as incorrect annotation and isoform coverage [13]. With the recent explosion in RNA-seq based genome wide analysis of the transcriptomic landscapes of multiple cell types and various tissues and organs, the time is ripe to use computational tools and a systems biology based approach to gain novel insights into tissue-specific gene signatures and their potential impact on biological processes.

In the present study, we have performed RNA-seq to generate a comprehensive gene expression profile of the mouse submandibular gland at various time points of development during embryogenesis and maturation in adult. To the best of our knowledge this is the first reported RNA-seq based study to examine the transcriptome of the mouse submandibular gland. An extensive bioinformatics analysis of our datasets has revealed interesting gene regulatory networks and maps that are enriched for and define the various stages of salivary glands. Finally we have leveraged the ENCODE, FANTOM, Human Protein Atlas as well as other published gene expression datasets to identify a salivary gland specific gene signature that is to a large extent conserved between mouse and human. Collectively our study not only validates existing literature but also provides a wealth of genomic resources that can be harnessed for the discovery of new genes and biologically important pathways in the salivary gland and for formulating testable hypotheses.

## Results

### Transcriptomic map of the mouse salivary gland during development and adult

**Fig. 1:**
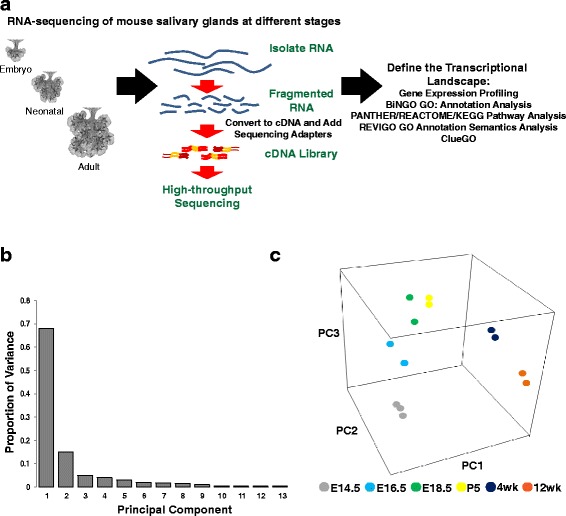
Principal component analysis of the mouse salivary glands at different developmental time points. **a** Experimental scheme. We isolated total RNA from whole salivary glands ranging from early embryo to adult, and performed RNA-seq. Utilizing these datasets, we defined and annotated the salivary gland transcriptional landscape by using various Gene Ontology (GO) annotation analyses (BiNGO GO, REVIGO GO) and pathway analyses (PANTHER/REACTOME/KEGG). **b** Proportion of variance in each principle component. PC1, PC2 and PC3 represent ~90% of variance in the data. **c** Projection plots show the PCA coordinates for each stage, which are indicated by different colors. The data indicates that the inherent variations in gene expression between biological samples can distinguish the developing salivary gland in a time dependent manner

In order to better define the dynamic changes in global gene expression levels and to identify new tissue-specific and tissue-enriched regulators, we performed RNA-seq based expression profiling of the mouse submandibular salivary gland at various key stages of embryonic development in addition to post-natal and adult tissues. Towards this end, we isolated total RNA from the mouse submandibular gland dissected from E14.5 day old embryos (Pseudoglandular stage - representing the onset of branching morphogenesis), E16.5 (Canalicular stage - representing the onset of cytodifferentiation) and E18.5 (Terminal Bud stage - representing the expansion of acini and lumenization of ducts and acini) [1, 4]. We also examined submandibular glands isolated from day 5 (P5), 4 week (4wk), and 12wk old male animals that represented different stages of post-natal maturation [1, 4]. Biological replicates of these RNA samples were sequenced to high depth using an Illumina HiSeq platform (Fig. 1a). The sequence reads were mapped to the reference genome sequence of *Mus musculus* (mm9 build) using Tophat2 (details in materials and methods). We subsequently performed between-sample normalization using the DESeq median normalization method and calculated fragments per kilobase of transcripts per million (FPKM) mapped reads thereby giving us measurements of relative expression of genes within and between biological samples.

In order to better analyze and appreciate the overall gene expression patterns between the various developmental and adult time points, we utilized principal component analysis (PCA), a statistical technique that reduces and summarizes large datasets while illustrating relationships between samples based on co-variance of the data being examined [14, 15]. Using PCA, we found that PC1, PC2, and PC3 accounted for approximately 90% of all variations of the original data (Fig. 1b). In order to further explore and better depict the major sources of variation, all samples were plotted in a three-dimensional space consisting of PC1, PC2 and PC3. Interestingly, as demonstrated in Fig. 1c, each of the 6 representative time points datasets segregated into individual groups demonstrating the highly dynamic variation in gene expression between each SG sample. Indeed, biological replicates cluster tightly together, further highlighting the inherent similarity of the biological samples to one another. Another notable observation was that the embryonic samples clustered more closely to each other and that there is a striking separation between these and the adult samples. Taken together this analysis provided the first hint of a clear dichotomy of gene expression profiles between embryonic and postnatal salivary gland samples.

### RNA-seq analysis identifies a salivary gland gene signature

To evaluate development-dependent differential gene expression patterns, we next grouped our samples based on 3 distinct developmental stages corresponding to embryogenesis (E14.5 to E18.5), neonatal (P5) and adult (4wk and 12wk). For this analysis, we selected genes that showed at least a two-fold change in expression between each time point while showing an adjusted *p*-value of less than 0.1. We also considered genes that were expressed at ≥1 FPKM in at least one biological replicate. Using this criterion, we identified a total of 3601 differentially expressed genes (DEGs) between the embryonic and neonatal stages with 1597 genes found to be up-regulated and 2004 genes showing downregulation (Additional file 1: Figure S1A). Similar comparison of DEGs between neonatal and adult developmental time points identified 3228 genes. Of these DEGs, 1281 were upregulated and 1947 downregulated (Additional file 1: Figure S1B). Finally, we found 5635 DEGs between embryo and adult with 2494 number of genes showing up-regulation and 3141 downregulated (Additional file 1: Figure S1C). To better appreciate the biological relevance of the global transcriptomic differences between the embryonic and adult mouse salivary gland, we further analyzed the DEGs using clusterProfiler [16] and identified pathways unique to each state (Additional file 1: Figure S1D). Interestingly, in all embryo enriched data sets, we observed specific enrichment of biological processes important during organogenesis, such as axon guidance as well as that of Wnt and Notch signaling pathways, both of which have been shown to be important for proper salivary gland morphogenesis [17–23]. In contrast, in the adult enriched data sets we observed enrichment in biological processes that include salivary secretion, protein export, fatty acid metabolism and vesicular signaling (Additional file 1: Figure S1D). Notably all of these processes have been shown to be critical for proper salivary gland function [24–27] and justifiably represent the mature stage of the adult gland.

**Fig. 2:**
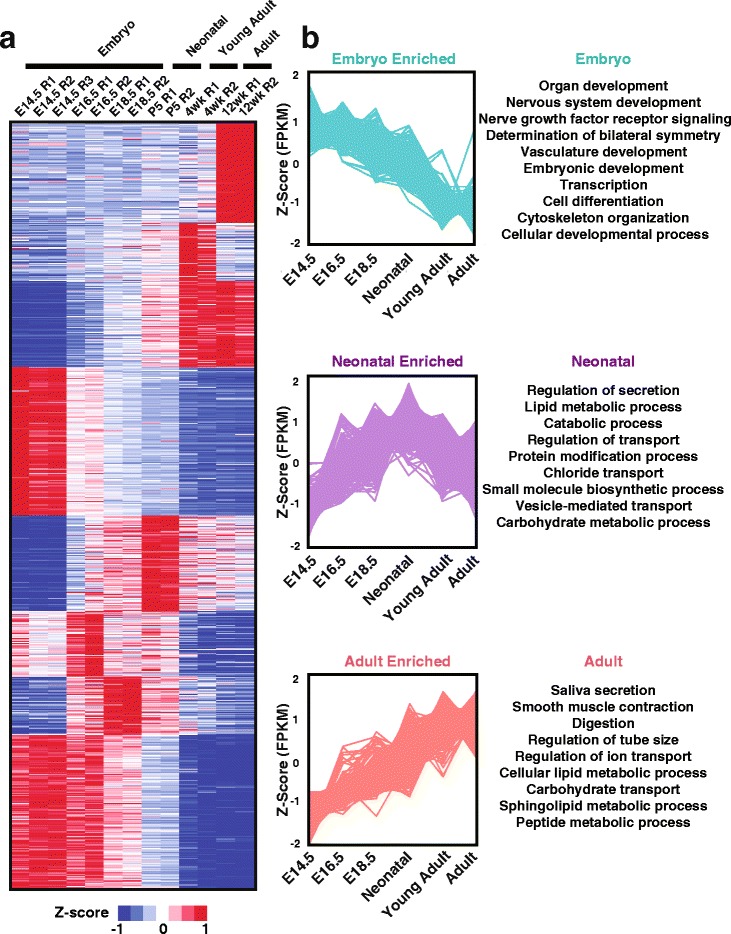
Cluster analysis of the salivary gland developmental profile. **a** Z-scores for each of the 1924 genes were calculated and used as input for gene-wise K-Means clustering analysis (k = 8, 1000 repetitions). This analysis, visualized as a heatmap, depicts enrichment of genes at both specific development time points and general developmental stages (embryo, neonatal and adult). **b** Visualization of the gene Z-scores from the general developmental stage enriched clusters depicts unique time-dependent patterns of expression. In general, this analysis has identified genes that have peak expression in the defined developmental stages. Also show are selected enriched GO-Annotations for each developmental stage gene cluster (BiNGO, Hypergeometric Test, FDR < 0.1)

Armed with a global view of the overall transcriptional changes occurring between the 3 developmental stages, we next sought to identify a developmental stage-specific global gene expression profile of the salivary gland. We reasoned that this broad gene profile might be useful in providing new insights into the biological processes and pathways that are important to each developmental stage. To generate the developmental specific gene expression profile, we focused on genes that a) showed an FPKM of 1 or greater in at least one of the sample replicates across all samples and b) were comparatively differentially expressed, with a log2-fold change of 1 or greater, at one time point compared to other time points across all 6 datasets. Based on the above criteria we identified a total of 1924 genes that were specifically enriched, across all datasets as demonstrated in Fig. 2a. To identify unique patterns of transcriptomic activities of the developmental stage-specific gene profile, we performed *K-*Means clustering on the standardized log2 transformed FPKM values for each of the samples. This analysis led to the identification of 8 different gene clusters that corresponded to genes enriched in a specific developmental time point as well as genes enriched in grouped time points, which we refer to as Embryo, Neonatal and Adult Enriched clusters (Fig. 2b). Interestingly, our analysis revealed that genes that were uniquely expressed during embryogenesis showed a gradual decline in expression as development progressed, while genes enriched in the neonatal stage showed a gradual increase in expression during embryogenesis with peak expression levels observed at the neonatal stage and then showing a gradual decline. Conversely, genes enriched in the adult stage showed a progressive increase during the developmental and neonatal stages with the highest levels of expression in the adult stage (Fig. 2b).

Gene Ontology (GO) analysis of the developmental stage-specific gene profile identified overrepresentation of genes associated with specific functions, indicating the existence of distinct biological processes occurring during embryonic, neonatal and adult stages of salivary gland development. A closer examination of genes that were uniquely expressed during embryogenesis revealed specific enrichment of biological pathways associated with organ, embryonic and vasculature development, cell differentiation, and transcription - all of these biological processes are associated with organ morphogenesis (Fig. 2b). Interestingly, we also observed specific enrichment in both nervous system development and nerve growth factor receptor signaling in salivary glands during embryogenesis. This is in good agreement with studies demonstrating the critical role of the parasympathetic nervous system during salivary gland organogenesis [20–22]. Genes specifically enriched in the neonatal stage also revealed association with interesting biological terms including regulation of secretion, lipid metabolic processes, catabolic processes, chloride transport, protein modification, and vesicular transport - all of which have been shown to play a role in secretory cell differentiation [24–26, 28–32]. Finally, analysis of the adult stage identified enriched biological pathways associated with salivary gland specific functions including saliva secretion, digestion, and ion and carbohydrate transport [25, 28, 33–35]. We also identified smooth muscle contraction as an enriched biological pathway in the adult SG (Fig. 2b), which fits very well with the existence of myoepithelial cell populations that are important for glandular contraction and expulsion of saliva. Taken together, the GO analysis, confirms the validity and the possible functional significance of our SG developmental stage-specific gene expression profile [36–38].

**Fig. 3:**
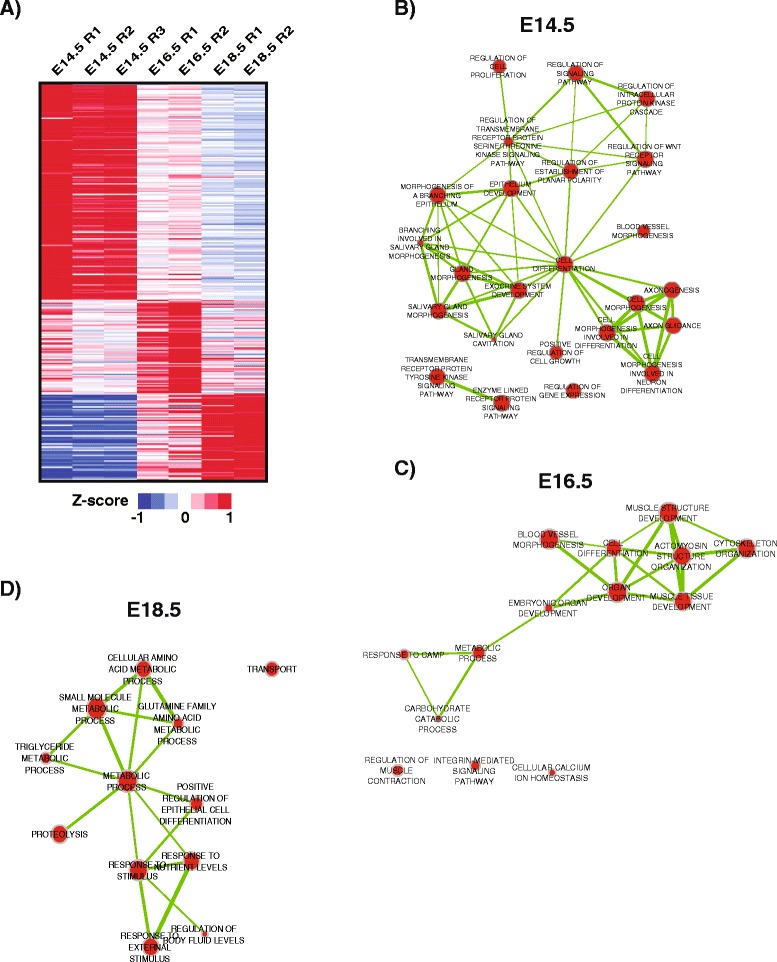
Enriched biological process networks during embryogenesis. **a** Heatmap visualization of the Z-scores from the 1064 genes identified in the embryo specific (E14.5, E16.5, E18.5) clusters generated from the analysis in Fig. 2. **b**–**d**) Network visualization of selected top enriched biological processes (BiNGO, Hypergeometric Test, FDR < 0.1) at E14.5, E16.5 and E18.5. The networks were assembled by CytoScape tool EnrichMap, using an organic layout. The node size represents the number of genes assigned to a biological process and edge width (*green line*) is proportional to the number of overlapping genes between two nodes

Since our analysis of the broad gene-enriched profile of embryonic, neonatal and adult salivary gland revealed interesting facets of its biology, we wondered if further in-depth parsing of the datasets could provide more detailed insights into salivary gland maturation, particularly in the embryonic stages. Hence, we next evaluated the transcriptomic activities from E14.5 to E18.5 to identify genes and pathways that may be critical during the early stages of salivary gland organogenesis and development. Of the 1924 comparatively differentially expressed developmental specific genes we identified, 1064 of them were specifically enriched during embryogenesis (Additional file 7: Table S1 and Fig. 3a, respectively). A closer analysis revealed that the greatest number of differentially expressed genes occurred at E14.5, at a stage when the gland is undergoing rapid proliferation and has embarked upon a dynamic and elaborate program of branching morphogenesis. While the E14.5 functional analysis demonstrated broad developmental processes including salivary gland morphogenesis, exocrine system development, epithelium development, axonogenesis and axon guidance, we also observed selective enrichment in biological processes specific to the developmental programs that are at play at this critical time point during morphogenesis. For instance, in agreement with the burgeoning branching morphogenesis that takes place at E14.5, genes involved in pathways specific to epithelial development, branching morphogenesis, and salivary gland cavitation were overrepresented (Fig. 3b). Indeed, both branching and cavitation are critical for the proper development of the tubular network necessary for the transport of saliva to the oral cavity [7, 8, 23, 39, 40]. While some degree of overlap was observed between E14.5 and E16.5 in processes involving cell differentiation and organ development, we did identify unique biological functions related to muscle function at E16.5. Interestingly, we found specific enrichment in pathways involving muscle structure, muscle tissue development and regulation of muscle contraction, properties intimately associated with functions of the myoepithelial cells [41, 42] (Fig. 3c). These findings are in good agreement with a previous study demonstrating the emergence of a myoepithelial cell population in the salivary gland at E16.5 [4]. Finally, our analysis at E18.5 uncovered several biological pathways enriched for a variety of salivary gland specific functions including metabolic processes (Fig. 3d). More specifically we observed over representation of metabolic processes important in amino acid metabolism and small molecule metabolism. We also identified pathways involved in transport and proteolysis all of which have been shown to play roles in salivary gland biology and secretion [43–47]. Taken together, our analysis suggests that the underlying global transcriptomic profile mirrors the distinct developmental stages of the embryonic SG as highlighted by branching morphogenesis (E14.5), myoepithelial development (E16.5) and secretory cell development (E18.5).

### Meta-analysis of mouse tissue RNA-seq expression data

**Fig. 4:**
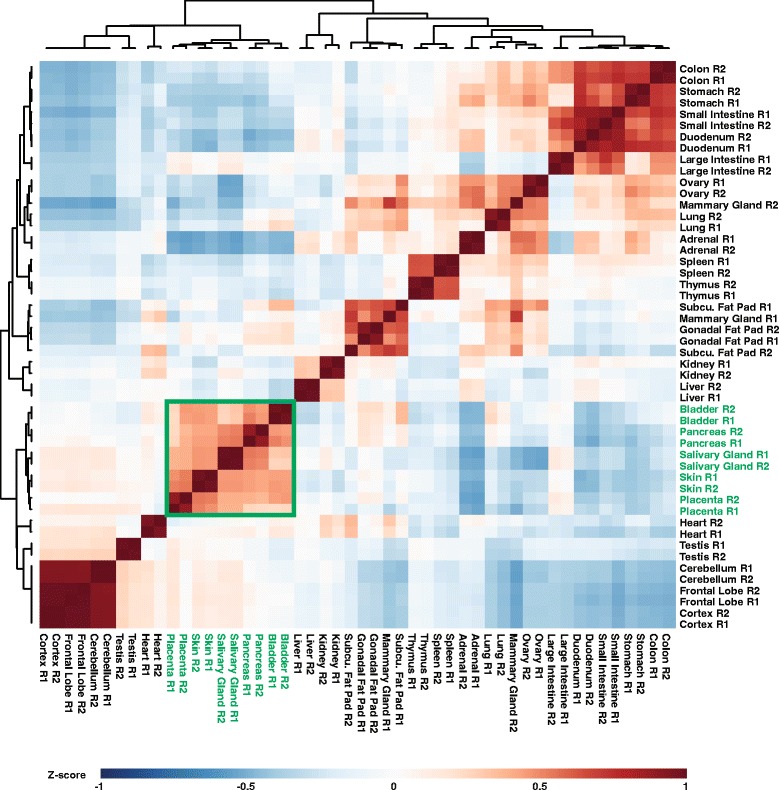
Hierarchical clustering of mouse tissues. FPKM values from the top 1500 genes with the highest median absolute deviation were used to cluster adult mouse tissues (Pearson Correlation, Average Linkage). The resulting heatmap shows that the salivary gland clusters closely with the pancreas, skin, bladder and placenta (*green box* and *text*)

Our salivary gland RNA-seq datasets offered a wealth of in-depth information regarding the transcriptomic repertoire of this tissue. We rationalized that although many of the genes and pathways are common to SG and numerous other tissues and organs, there might exist some genes/pathways that are enriched for in expression and possibly function in the SG. To probe this further, we examined RNA-seq data for 24 adult organs and tissues generated by the mouse ENCODE consortium [48], and compared the overall gene expression profiles with those from the adult SG described in this study. For this purpose, we generated a pairwise Pearson Correlation matrix using the top 1500 genes with the highest median absolute deviation. This, we reasoned, would allow us to identify genes with the most dynamic range of expression across all tissues. Our analysis revealed a clear separation of organs based on gene expression with the salivary gland clustering more closely with exocrine organs such as the pancreas and organs rich in stratified epithelial tissues including the skin, bladder and placenta (Fig. 4, green box). The organ-specific segregation patterns were not a result of using a stringent cutoff of the top 1500 genes, since a pairwise Pearson Correlation matrix using a larger set of 19,272 genes, yielded similar results with the salivary gland continuing to cluster closely with the pancreas, skin, bladder and placenta (Additional file 2: Figure S2, green box). A similar hierarchical clustering analysis also revealed a select list of genes that are similarly expressed between the salivary gland and the closely related four organs identified above (Additional file 3: Figure S3A and Additional file 7: Table S2) and those that are exclusive to the two exocrine glands; the salivary gland and pancreas (Additional file 3: Figure S3B and Additional file 7: Table S3). Interestingly, the transcriptomic identity of the mouse salivary gland was quite distinct from the mammary gland, another glandular tissue. We suspect this is due to the fact the secretory activity of the mammary gland is limited only during parity and lactation rather than the virgin state, from which the RNA-seq data was obtained. In contrast, tissues representing specific brain regions such as the cerebellum and the mouse testis were surprisingly clustered close to the group containing the salivary gland and the four aforementioned organs – the significance of this finding is unclear and worthy of future exploration. Taken together, our findings demonstrate that at the broader global transcriptomic level, the salivary gland most closely resembles tissues and organs with similar morphological and functional characteristics.

**Fig. 5:**
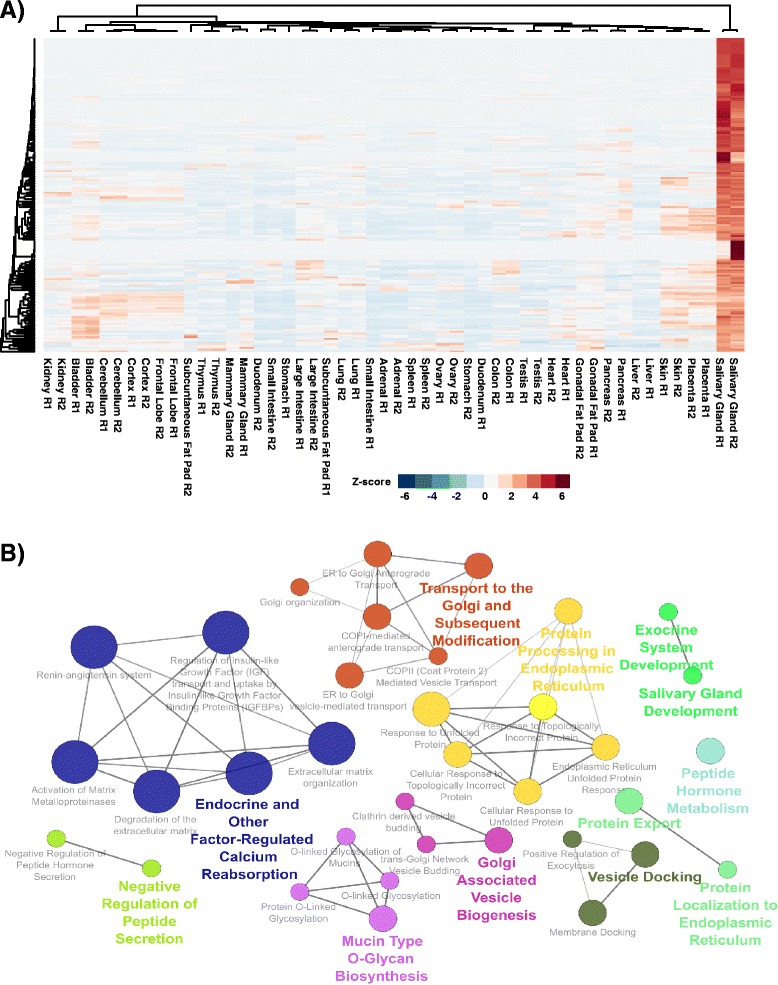
Visualization of the tissue specific salivary gland gene signature. **a** Hierarchical Clustering (Pearson Correlation, Average Linkage) of the gene expression values selected from the tissue specific salivary gland signature across adult mouse tissues. **b** Network visualization of enriched pathways (GO/REACTOME/KEGG) in the gene signature was performed by ClueGO analysis

Having obtained a global gene expression profile of the salivary gland, we next wondered if we could derive an adult salivary gland specific molecular signature that is constituted by genes that show a distinct expression profile and hence are likely to be more relevant and important in salivary gland biology. Towards this end, we next compared gene expression levels across all adult mouse organs and tissues and identified 174 genes that were specifically enriched in the salivary gland and thus represented a potential salivary gland gene signature (Fig. 5a and Additional file 7: Table S4). To gain a better understanding of the underlying biological functions and pathways associated with the adult salivary gland gene signature, we utilized ClueGO [49], which is a functional gene ontology analysis tool that integrates several gene-set enrichment databases, including the Kyoto Encyclopedia of Genes and Genomes (KEGG), REACTOME pathway database annotations, and the GO Consortium database, to create a comprehensive GO/pathway term network [49]. This analysis not only highlighted the close association of the signature to exocrine and salivary gland development but also identified enrichment in biological processes related specifically to protein export, protein processing and localization to the endoplasmic reticulum, all of which are quite relevant for proper salivary gland function (Fig. 5b). Interestingly, we also observed pathway enrichment specific to mucin biosynthesis – this is in good agreement with the established roles of mucins in salivary gland biology that includes their role as a first line of defense against microbial infection and their important contributions to various biophysical properties of saliva such as viscoelasticity [50, 51].

**Fig. 6:**
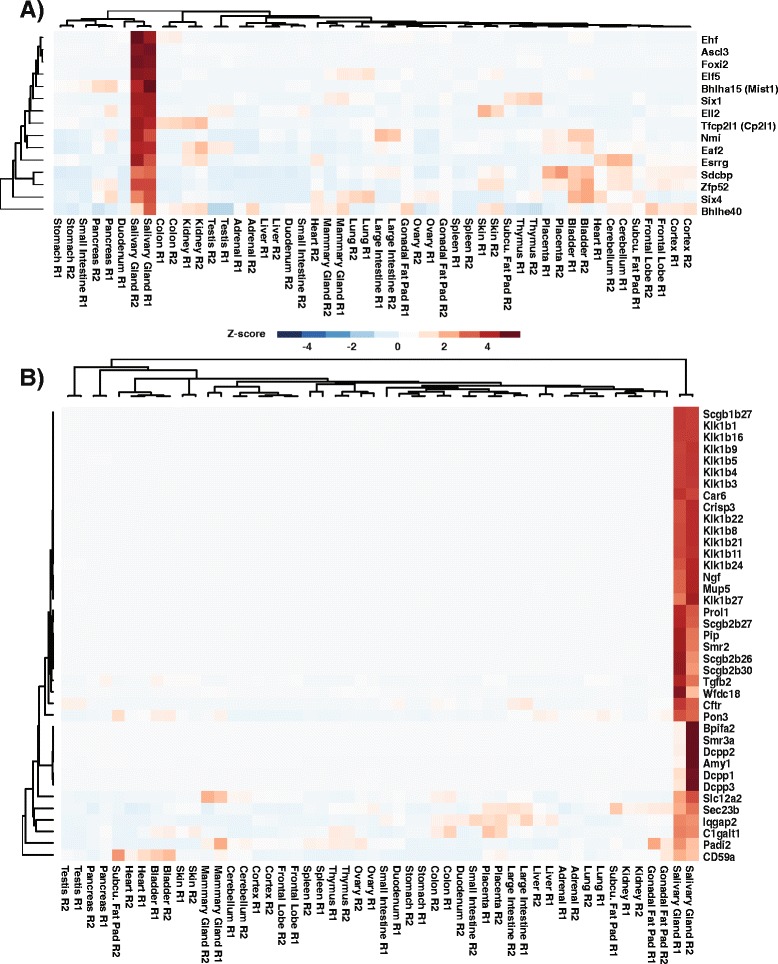
Visualization of selected members of the tissue specific salivary gland gene signature. **a** Hierarchical cluster analysis of the enriched transcription factors identified in the gene signature. **b** Hierarchical cluster analysis of enriched genes identified in the signature and which have been shown to play a role in salivary gland biology

We next sought to identify a cohort of transcription factors, which are selectively enriched in our SG gene signature. Based on the crucial regulatory role transcription factors play in driving tissue-specific gene expression, we reasoned that this analysis may aid in identifying novel transcriptional regulators which may be important in salivary gland biology. To ensure robust coverage, we first compiled a comprehensive mouse transcription factor list using databases from both RIKEN [52] and UniProtKB and then searched for transcription factors which are represented in our mouse adult salivary gland gene signature. Our analysis uncovered 15 transcriptional regulators (Fig. 6a and Additional file 7: Table S4). Of these transcription factors, Ascl3 [53, 54], Bhlha15 (Mist1) [55, 56], Tfcp2l1 (Cp2l1) [57] and Six1 [58] have been previously reported to play a role in salivary gland development and function. Our analysis also unearthed Elf5 [59] and Ehf [60], two members of the Ets family of transcription factors, which are highly expressed in the salivary gland yet their specific function, if any, in salivary gland biology remains unknown. Moreover, we identified several other factors including Eaf2 and Foxi2 neither of which has been previously studied in the context of salivary gland biology. The high expression of some of these transcription factors in salivary glands was also confirmed using publicly available datasets provided through the Salivary Gland Molecular Anatomy Project (Additional file 4: Figure S4). In addition to the list of transcriptional regulators that were specifically enriched in our salivary gland gene signature, there were several other genes and gene family members that are both over-represented and likely or known to be associated with salivary gland biology (see heat map Fig. 6b). This included genes belonging to the secretoglobin (SCGB) gene superfamily, which encode for small secretory proteins found in high concentrations in secretions from various organs including the lacrimal and salivary glands [61]. In addition, we observed selective enrichment in genes belonging to the Kallikrein (KLK) family that encode for proteins which function as serine peptidases. While a major function of this family of proteins have been associated with blood pressure regulation and skin homeostasis, some studies suggest that these enzymes may play a role in cell proliferation, cell survival and wound healing [62]. Finally, we also observed elevated levels of genes belonging to the Demilune Cell and Parotid Protein (DCPP) family, which are a family of genes thought to possess antimicrobial activity [63]. We posit that many of these genes unearthed by our systems biology based approach are likely to be strong candidates for various physiological functions associated with mouse salivary glands.

### Evolutionarily conserved expression map between human and mouse salivary gland

**Fig. 7:**
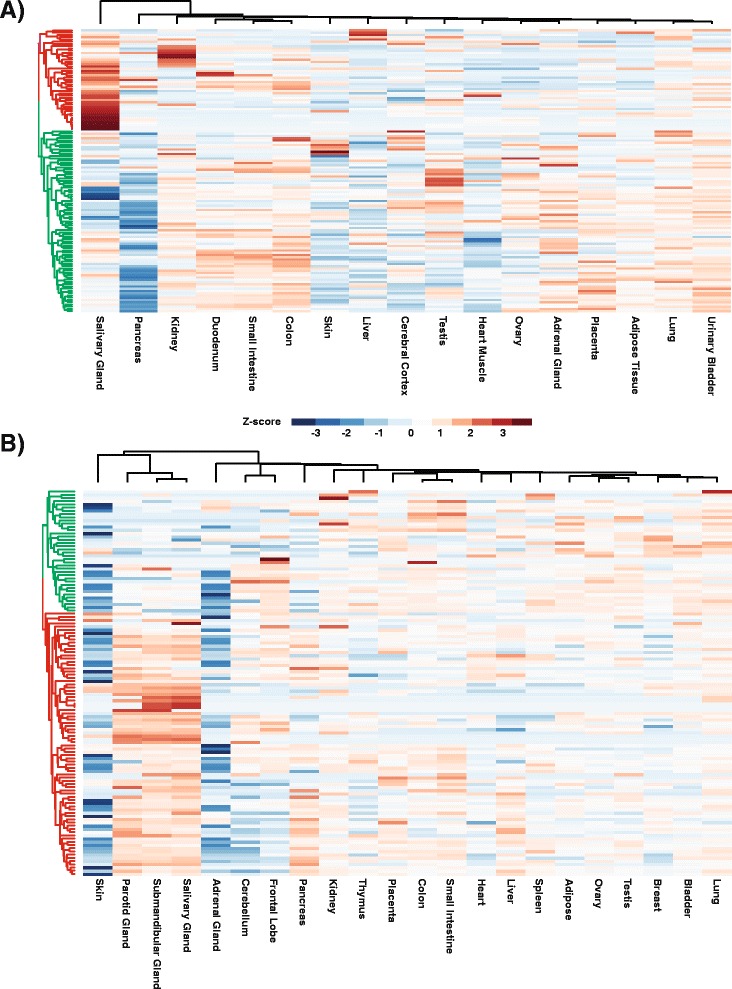
Preservation of the tissue specific salivary gland gene signature in human tissues. **a** Hierarchical clustering of human tissues using averaged FPKM values (Human Protein Atlas RNA-seq Experiments) of the genes from the tissue specific salivary gland gene signature with human-mouse homology. The *red colored dendrogram* highlights the genes (45/126) that are preserved in the tissue specific enrichment observed in the mouse expression analysis. **b** Hierarchical clustering of human tissues using the Cap Analysis Gene Expression (CAGE) data, represented by log2 transformed DESeq2 median normalized TSS tag counts using the same gene signature as in panel A, with 82 genes showing cross-species conservation (*red colored dendrogram*)

Mouse models have served a valuable role in elucidating molecular mechanisms of physiological and pathological processes in the salivary gland. It is likely that the genetic interplays and gene regulatory networks that underlie these processes are mostly conserved between mouse and human. To examine this we next asked if there were any commonalities in the salivary gland specific gene signature between mouse and human. Towards this end, we mined the publicly available transcriptome database for human tissues and organs generated by the Human Protein Atlas (HPA) project (Illumina-based RNA-seq) and the FANTOM5 consortium (Heliscope-based CAGE (Cap Analysis Gene Expression)). To make proper comparisons, we only chose representative human tissue samples for which the corresponding data was available for the mouse tissues. Upon examination of the Human Protein Atlas (HPA) samples, we found that of the 126 human genes that were homologous to the mouse, 45 genes (~36%) showed enrichment in the human salivary gland as compared to the 16 other tissues analyzed (Fig. 7a, Additional file 5: Figure S5 and Additional file 7: Table S5), thus recapitulating the tissue-restricted patterns of expression observed from our mouse tissue analysis. One possible caveat to this comparison is that for the HPA dataset, there is limited information about the salivary gland, specifically its subtype or the age of the donor. The CAGE tissue transcriptome from RIKEN on the other hand, contained not only representative adult human submandibular glands but also parotid glands thus allowing a more equitable comparison. Upon hierarchical clustering of the processed CAGE data (see methods for details), the preservation of the mouse tissue specific signature was distinctly more robust, with 82 genes (~65%) showing cross-species conservation based on shared levels of salivary gland specific gene enrichment (Fig. 7b, Additional file 6: Figure S6 and Additional file 7: Table S6). Taken together, our analysis point to a conserved network of crucial genes and pathways that regulate common physiological processes in mouse and human SG and underscores the overall value and usefulness of mouse genetic models to study this tissue.

## Discussion

Proper organ development requires remarkably intricate and complex biological processes that rely on spatially and temporally controlled gene expression programs. This paradigm is quite apparent in the case of the submandibular salivary gland, which undergoes a dynamic process of morphogenesis during embryogenesis and further specification and functional maturation after birth. In order to better understand the molecular underpinnings and identify novel and unique genes that may play important roles in salivary gland biology, here we have performed genome wide expression profiling of the mouse salivary gland during various stages of development and adult. In addition to generating stage-specific gene expression profiles of the submandibular salivary gland, we have also utilized sophisticated computational tools and leveraged the large ENCODE and FANTOM based data sets to obtain a systems biology driven perspective on this complex organ.

We have focused our studies on 3 different embryonic time points during which the salivary gland undergoes tremendous growth and morphological changes including branching morphogenesis, cyto-differentiation and lumenization. As expected, these changes are associated with distinct alterations in the gene expression profile of critical regulatory factors and pathways, some of which are likely to be critical drivers of the developmental processes. Our analysis has led to the identification of a stage-specific and more broadly embryonic-specific gene expression profile, which can serve as a starting point in identifying new players that may be important in salivary gland development. For example after mining our embryo enriched dataset, we identified several genes which showed peak expression levels very early during salivary gland morphogenesis and then declined over the course of late embryonic development and into adult. While several of the highly differentially expressed genes at E14.5 have been implicated in axonal guidance such as Unc5d [64] and Tubb2b [65], we also identified additional genes from this group that may be interesting for future follow up studies including Scube1 [66] and Dlx1 [67], both of which have been implicated in craniofacial development but not in salivary gland biology. Needless to say, RNA-seq based analysis similar to those described in this work can be further extended to additional time points during mouse embryogenesis, in particular at stages earlier than E12.5 during which the salivary gland fate is being established.

Our RNA-seq based experiments not only allowed us to generate a broad transcriptomic map of the postnatal salivary gland, but also yielded interesting insights into the continued salivary gland development and maturation processes that occur after birth. Indeed, our analysis demonstrated elevated expression levels of genes important in secretory cell differentiation during early postnatal development further highlighting the sustained cellular differentiation programs that continue after birth. In contrast, at ~12 weeks the adult salivary gland is enriched for genes that serve valuable functional roles in this organ. Among these, we identified Amy1 as one of the most highly expressed genes, which is in good agreement with the known function of this gene in salivary gland biology and oral health [68]. We also observed specific enrichment of the solute carrier family of genes (Slc) which have been shown to be important in fluid and electrolyte secretion in the salivary gland [69].

A major innovative aspect of our study is the development of the adult salivary gland gene signature – we posit that the unique combination of genes bestows an organ its tissue identity and can be extremely revelatory about organ development and function. By leveraging ENCODE data and using robust statistical analyses, we have demonstrated that at the global transcript levels, the adult salivary gland most closely resembles another exocrine organ, the pancreas as well as several other epithelial-rich organs including the bladder, skin and placenta. Although these global transcriptomic comparisons are per se quite insightful and suggest common developmental origins or functional similarities between organs, caveats such as differences in age, sex and the physiological state of the animals and the experimental conditions, is worth keeping in mind. As an example, unlike the tight clustering of the pancreas and salivary gland, the mammary gland exhibited a slightly distant gene expression signature. We suspect that this lack of similarity might be in part due to the fact that the secretory activity of the mammary gland is primarily limited to lactation – a physiological state that was not included in our organ comparisons. Our analysis also allowed us to generate a list of genes that are highly enriched for in the mouse salivary glands, a subset of which also show similar selective enrichment in the human salivary gland. Notably these salivary gland-enriched genes hold the key to crucial aspects of adult salivary gland biology as evident from Gene Ontology terms and thus offer a priority list of candidates that can be leveraged for salivary gland specific functions. For example in a recent study, Maruyama et al. generated three inducible Cre-recombinase mouse strains to examine the roles of various cell types in salivary gland homeostasis - it is interesting to note that all three genes examined in this study are included in our salivary gland gene signature analysis [70]. Moreover, the datasets generated by our RNA-seq studies can serve as a nice complement to other resources such as the rapidly expanding EMAGE (e-Mouse Atlas of GeneExpression) database of in situ gene expression in the mouse embryo [71]. As an example, a cursory examination of EMAGE showed strong expression of the transcription factor Ehf in the submandibular gland primordium, in agreement with its high expression as revealed from our RNA-seq analysis. Therefore, our salivary gland gene signature can be a valuable tool for future studies on cell/tissue type specific gene expression mechanisms or delivery tools for salivary gland biologists.

## Conclusion

Our work as described here is the most comprehensive systems-wide deep sequencing based transcriptomics study of the submandibular salivary gland to date and illustrates how such studies can shine new light into the rich diversity of genes and pathways that are likely to be functionally important for this organ. Knowing the gene expression profiles of the salivary gland at its major developmental time points can greatly increase our understanding of salivary gland biology, aid with disease state diagnosis, and help identify potential therapeutic targets for regeneration and tissue engineering approaches in the future.

## Methods

### Animal experiments

All animal experiments were performed in compliance with Roswell Park Cancer Institute IACUC regulations. C57BL/6 mice were mated and noon of the day the vaginal plug was observed was considered E0.5. Submandibular glands were dissected from animals at specific embryonic and adult time points. Due to limited amounts of tissues, biological replicates for E14.5, E16.5 and E18.5 were generated by pooling glands from 3, 2, and 2 animals, respectively. The biological sex of the animals was not ascertained for the embryos and P5 mice. Analysis of 4 and 12 week old salivary glands was performed using male mice.

### RNA isolation and RNA-seq analysis

Total RNA was extracted using the Direct-zol RNA MiniPrep kit (Zymo Research), from dissected mouse C57BL/6 submandibular glands at indicated time points. For each RNA sample, cDNA libraries were prepared using the TrueSeq RNA Sample Preparation Kit (Illumina) and were then 50 bp single-end sequenced on an Illumina HiSeq 2500. Quality control metrics were performed on raw sequencing reads using the FASTQC v0.4.3 application. Reads were mapped to the Mus musculus genome (mm9 build) with TopHat2 [72] v2.0.13, using Bowtie2 [73] v2.2.6 as the underlying aligner. Reads aligning to the UCSC mm9 build were quantified with featureCounts [74], which disregarded any read/read pair that aligned to more than one location, or more than one gene at a single location. DESeq2 [75] was used to normalize the read counts and derive FPKM values. MouseENCODE [76] Raw RNA-seq experiments of adult male whole tissue from the CSHL Long RNA-seq dataset as well as the Synder dataset were downloaded as fastq files and processed as paired end experiments identically to the in-house generated data. The adult mouse skin tissue RNA-seq experiments were previously generated by our lab and reprocessed [77].

### Bioinformatics analysis

#### Generation of the development specific salivary gland gene profile

All differential gene expression (DEG) analysis was performed using DESeq2. The developmental stage salivary gland count data was imported as one matrix and was subsequently divided into two separate DEG analyses. The first analysis treated each of the embryo time-points as biological replicates as well as the two separate adult stages when making contrasts as part of the DESeq2 standard protocol. Genes were considered differentially expressed if the log2 Fold Change between samples was at least 1, with the adjusted *p*-value held to 0.1. Additionally we considered only genes with values of at least one FPKM in at least one biological replicate. The second analysis involved the creation of the salivary gland developmental gene profile. In this analysis, all time-points were considered separate and only true biological replicates were used. In order to create a highly stringent list of time-point specific genes the following approach was used: 1) Contrasts were created between one time-point and every other subsequent time-point 2) DEGs for each of these analyses were called using the aforementioned criteria and 3) The intersection of these DEGs were reported for the individual time-point, thereby creating a stringent list of genes that were consistently differentially expressed in either the positive or negative direction. This process was iterated until all time-points were analyzed identically. The union of genes identified in the aforementioned analyses were taken and reported as the gene profile for the developing salivary gland. K-Means clustering was used to capture the patterns of gene expression as development proceeds in the salivary gland. Gene FPKM values were averaged across biological replicates and subsequently standardized to their Z-Scores. These values were used as inputs to the K-Means clustering algorithm (cluster3.0 [78]) using k = 8 clusters.

#### Development of a tissue specific salivary gland gene signature

Counts data from the MouseENCODE data plus the whole adult skin and the 12 week old salivary gland (12wk SG) experiments were imported and normalized as one matrix. In order to identify gland specific genes, DESeq2 was used to create contrasts between the 12wk SG and each of the 24 other tissues. DEGs were reported for each contrast according to the aforementioned criteria and the intersection of the genes from each analyses that were considered differentially expressed in the positive direction (i.e. more highly expressed in salivary gland) were reported as the tissue specific salivary gland gene signature.

### Hierarchical cluster analysis

All analyses and figures were made using R v3.2.3. The heatmap program from the NMF [79] v0.20.6 R-package was used to generate heatmaps. The correlation plot from Fig. 4 was generated by performing pairwise Pearson Correlations on the log2(FPKM + 1) values of the top 1500 genes with the highest median absolute deviation. The resulting matrix was reordered using hierarchical clustering, using Pearson Correlation as the distance measure with average linkage. All subsequent gene-wise and sample-wise hierarchical clustering analysis (Figs. 4, 5, 6 and 7) was performed using Pearson Correlation with average linkage, after which the resulting reorganized data matrix was transformed row-wise to report Z-scores of each gene across samples for heat-map visualization purposes.

### Preservation of the mouse tissue specific salivary gland gene signature in human

#### Human protein atlas

In order to examine if the same genes identified in the tissue specific salivary gland signature would be enriched in human salivary gland compared to all other tissues, processed RNA-seq experiments generated by the Human Protein Atlas [80] were downloaded as FPKM values that were averaged across biological replicates. Only human organs and tissues corresponding to those used in generating the mouse specific signature were selected and only genes that are homologous between mouse and human, as identified by the BioMart [81] data mining tool of the Ensembl database, were used for subsequent analysis. Hierarchical clustering analysis was performed on the 126 genes that met this criteria and the cluster of genes that shared similar expression patterns as compared to the mouse analysis was identified using the dendextend [82] v1.1.2 R-package.

#### FANTOM CAGE analysis

The R-package CAGEr(v1.12.0) was used to download raw FANTOM5 Cap Analysis of Gene Expression (CAGE) data taken from human tissue samples. Only samples which matched those used in the mouse specific analysis were selected. Raw tag counts were read in for each sample and TSS locations were estimated using the clusterCTSS function, which will cluster neighboring TSSs if they are closer than 20 bp and remove potential TSS sites if there are less than 2 tag counts. Promoter widths were calculated by calculating the cumulative distribution of CAGE tag signal across the clustered TSS locations, followed by determining the position of the Upper and Lower quantile locations of the cumulative CAGE signal, whereupon the distance between these quantiles represents the promoter width. Consensus promoter locations across all samples were determined in order to best estimate gene expression. The consensusCluster functions was used to aggregate tag clusters using the promoter widths calculated previously, whereupon promoter locations were merged if located within 100 bp of each other and TSS locations with extremely low overall tag counts were eliminated. This provided a raw count matrix for all determined TSS locations, which represented overall gene expression. Nearest gene annotation was applied for each TSS location and promoter tags were aggregated based on gene identity. This gene-level tag count matrix was then used as input for DESeq2 differential gene expression analysis pipeline. This final count matrix was log2 transformed, DESeq2 median normalized and filtered for generation of the mouse tissue specific gene signature.

### GO annotation and enrichment

The KEGG enrichment analysis shown in Additional file 1: Figure S1 was carried out using the R package clusterProfiler [16] v2.4.1. Subsets of genes identified by DEG analysis carried out between developmental groups were binned into lists representing genes that were enriched in expression for the specific time point. These lists were independently used as input to the enrichKEGG. Over-represented gene sets were identified with Hypergeometric Testing using the Benjamini-Hochberg method (α = 0.1) to control for False Discovery Rate (FDR). GO Annotation enrichment for biological processes found in Figs. 2 and 3 were generated using BiNGO [83]. The resulting output was ranked by adjusted *p*-value and gene-sets were selected to highlight unique features of the enrichment analysis. These features were graphically represented using the EnrichmentMap [84] tool, which is an add-on application for Cytoscape [85], using default parameters. KEGG, REACTOME Pathways and GO Enrichment Analysis was performed using the ClueGO [49] add-on for the Cytoscape Platform. The tissue-specific salivary gland signature was used as the input for the analysis, which was performed using default parameters.

## Additional files

Additional file 1: Figure S1. Differential gene expression analysis of the developing salivary gland. (A-C) Scatter plots of log2 fragments per kilobase of transcripts per million (FPKM) mapped read values between different developmental stages are shown. Red points indicate genes that are enriched in the sample on the Y-axis, and blue points show genes that are enriched in the sample on the X-axis. Black points highlight transcription factors that are upregulated in their respective samples. D) Enriched biological pathways (KEGG) of differentially expressed gene identified in panels A-C above. Analysis was performed using clusterProfiler package. (PDF 2353 kb)
Additional file 2: Figure S2. Hierarchical clustering of mouse tissues. FPKM values from 19,272 genes were standardized and subsequently used to cluster adult mouse tissues (Euclidean Distance, Complete Linkage). The resulting heatmap shows that the salivary gland clusters closely with the pancreas, skin, bladder and placenta (green box and text). (PDF 998 kb)
Additional file 3: Figure S3. Hierarchal clustering of the common genes expressed in salivary gland, pancreas, skin, bladder and placenta. A) Clustering of genes that collectively show higher expression in the salivary gland, pancreas, skin, bladder and placenta in comparison to all other tissues. B) Clustering of highly expressed genes shared between the two exocrine glands, the salivary gland and pancreas, in comparison to all other tissues. (PDF 11034 kb)
Additional file 4: Figure S4. Confirmation in expression of select transcription factors which comprise the adult salivary gland gene signature. Probe intensity levels of a subset of transcriptions factors which make up the salivary gland gene signature, as provided through the Salivary Gland Molecular Anatomy Project, are shown. Various stages of development and adult are included. P1- postnatal day 1, P5- postnatal day 5, AdF – adult female, AdM- adult male. (PDF 1355 kb)
Additional file 5: Figure S5. Heatmap depicting the hierarchical clustering of the 45 genes that are conserved between the mouse adult salivary gland gene signature and the RNA-seq data obtained from the Human Protein Atlas. The values reported represent Z-scores of the conserved genes in their respective datasets. (PDF 737 kb)
Additional file 6: Figure S6. Hierarchical clustering of the 82 genes that are conserved between the mouse adult salivary gland gene signature and the Cap Analysis Gene Expression (CAGE) datasets. The human submandibular gland is shown as a comparison. The values visualized in this heatmap represent standardized expression levels of the selected conserved genes relative to their respective datasets. (PDF 751 kb)
Additional file 7: Table S1. Developmental gene signature. **Table S2.** Common genes expressed between salivary gland, pancreas, skin, bladder and placenta. **Table S3.** Common genes expressed between salivary gland and pancreas. **Table S4.** Adult gene signature. **Table S5.** HPA data. **Table S6.** Human CAGE data. (XLSX 80 kb)

## Abbreviations

BMP: Bone Morphogenetic Protein
CAGE: Cap Analysis Gene Expression
DEG: Differentially expressed genes
EMAGE: e-Mouse Atlas of Gene Expression
ENCODE: Encyclopedia of DNA elements
FGF: Fibroblast Growth Factor
FPKM: Fragments per kilobase of transcripts per million
GO: Gene ontology
GSK3β: Glycogen synthase kinase 3-beta
HPA: Human Protein Atlas
KEGG: Kyoto Encyclopedia of Genes and Genomes
NGS: Next generation sequencing
PCA: Principle component analysis
RNA-seq: RNA-sequencing
SG: Salivary gland
SMG: Submandibular gland
TSS: Transcription start site
