# Supplementary figures and images for: RNA-seq based transcriptomic map reveals new insights into mouse salivary gland development and maturation
Source: BMC Genomics. 2016 Nov 16;17:923. doi: 10.1186/s12864-016-3228-7 (PMC5112738; doi:10.1186/s12864-016-3228-7)

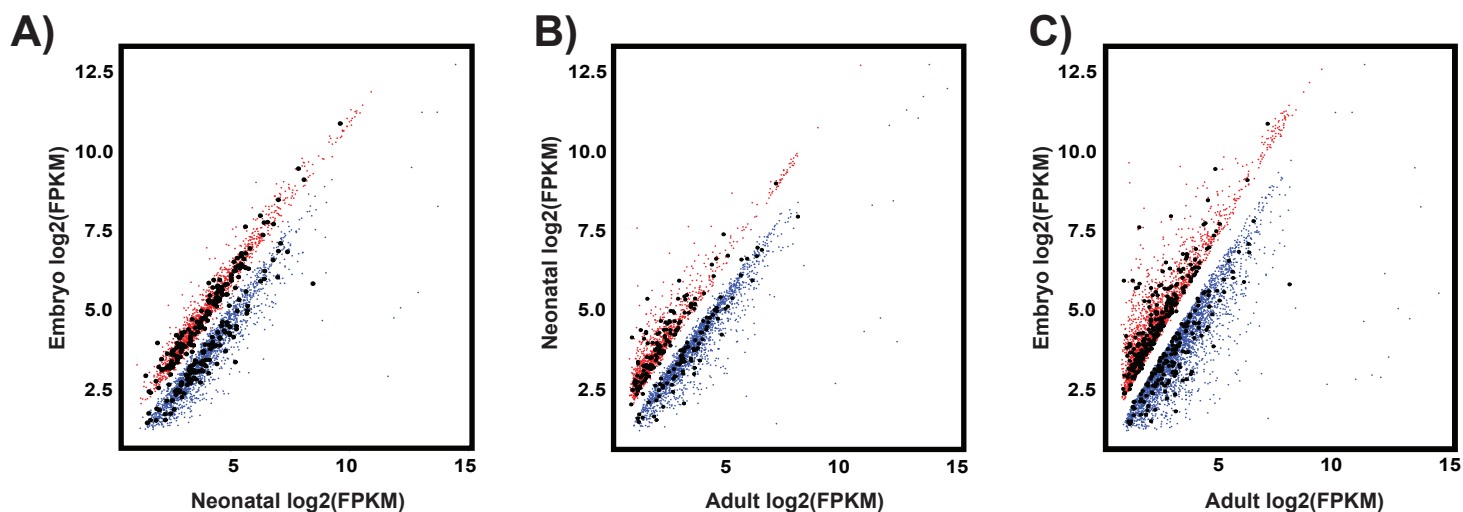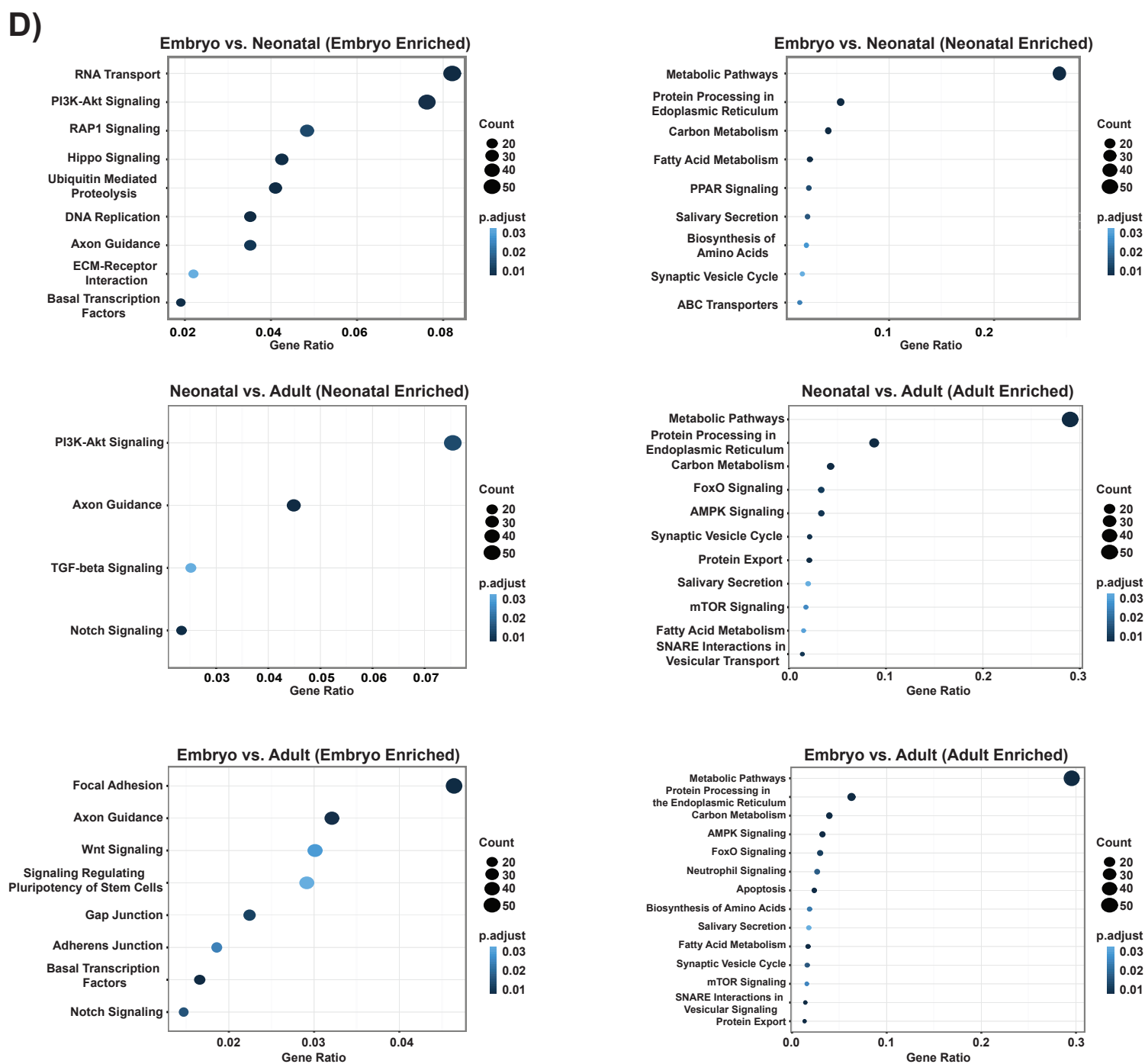

Supplement: Additional file 1: Figure S1. — Differential gene expression analysis of the developing salivary gland. (A-C) Scatter plots of log2 fragments per kilobase of transcripts per million (FPKM) mapped read values between different developmental stages are shown. Red points indicate genes that are enriched in the sample on the Y-axis, and blue points show genes that are enriched in the sample on the X-axis. Black points highlight transcription factors that are upregulated in their respective samples. D) Enriched biological pathways (KEGG) of differentially expressed gene identified in panels A-C above. Analysis was performed using clusterProfiler package. (PDF 2353 kb) [file 12864_2016_3228_MOESM1_ESM.pdf]

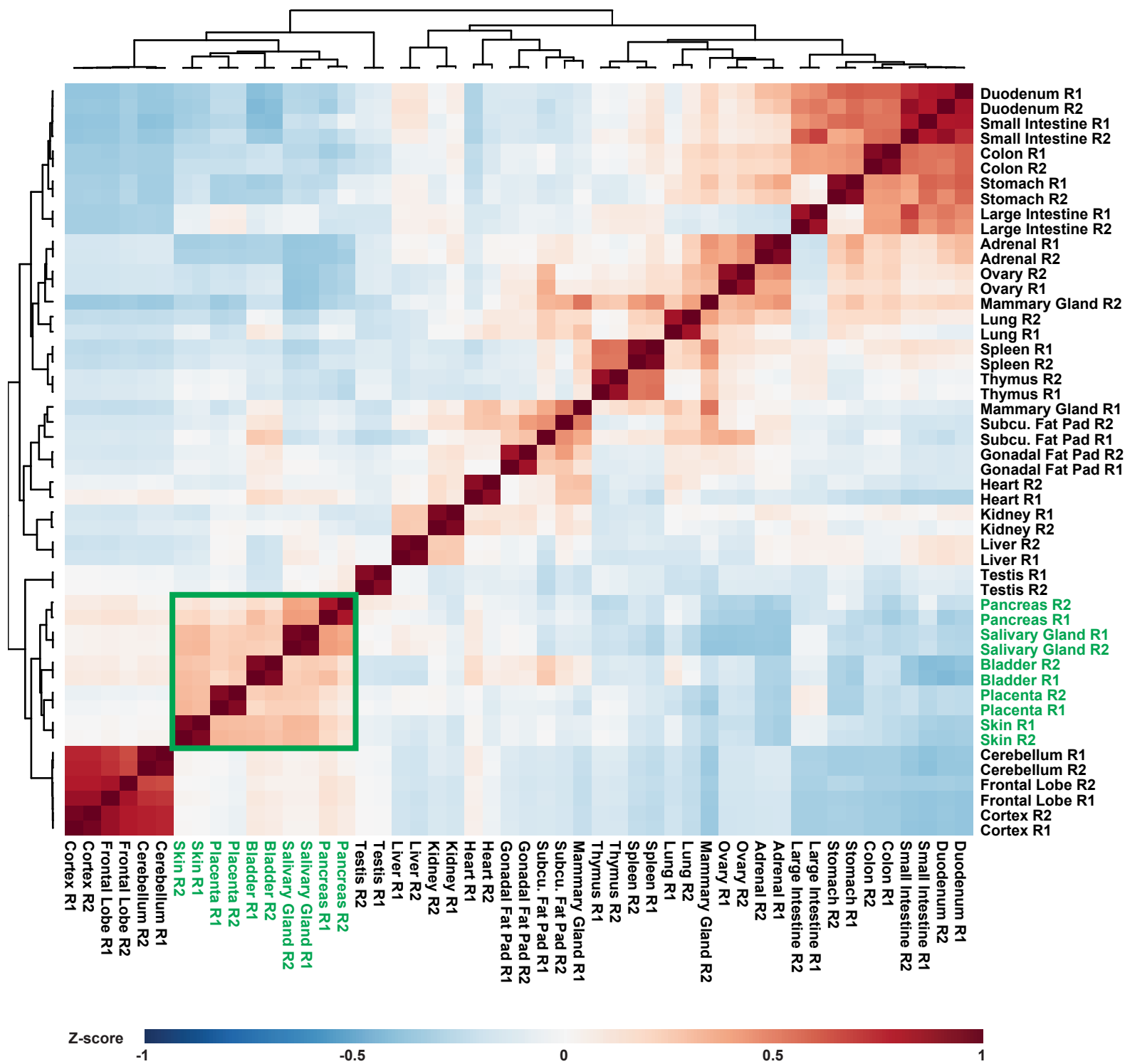

Supplement: Additional file 2: Figure S2. — Hierarchical clustering of mouse tissues. FPKM values from 19,272 genes were standardized and subsequently used to cluster adult mouse tissues (Euclidean Distance, Complete Linkage). The resulting heatmap shows that the salivary gland clusters closely with the pancreas, skin, bladder and placenta (green box and text). (PDF 998 kb) [file 12864_2016_3228_MOESM2_ESM.pdf]

A)

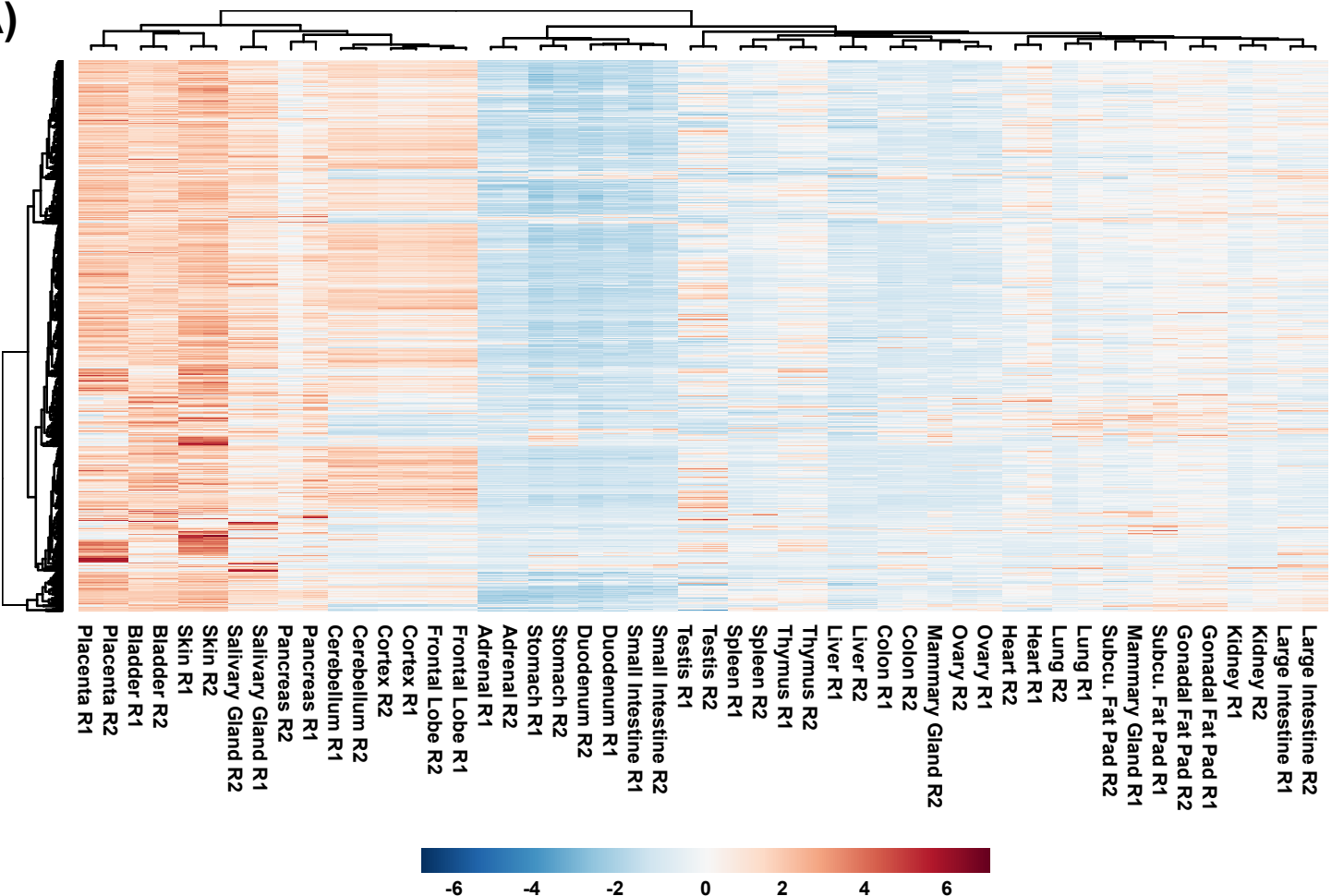

B)

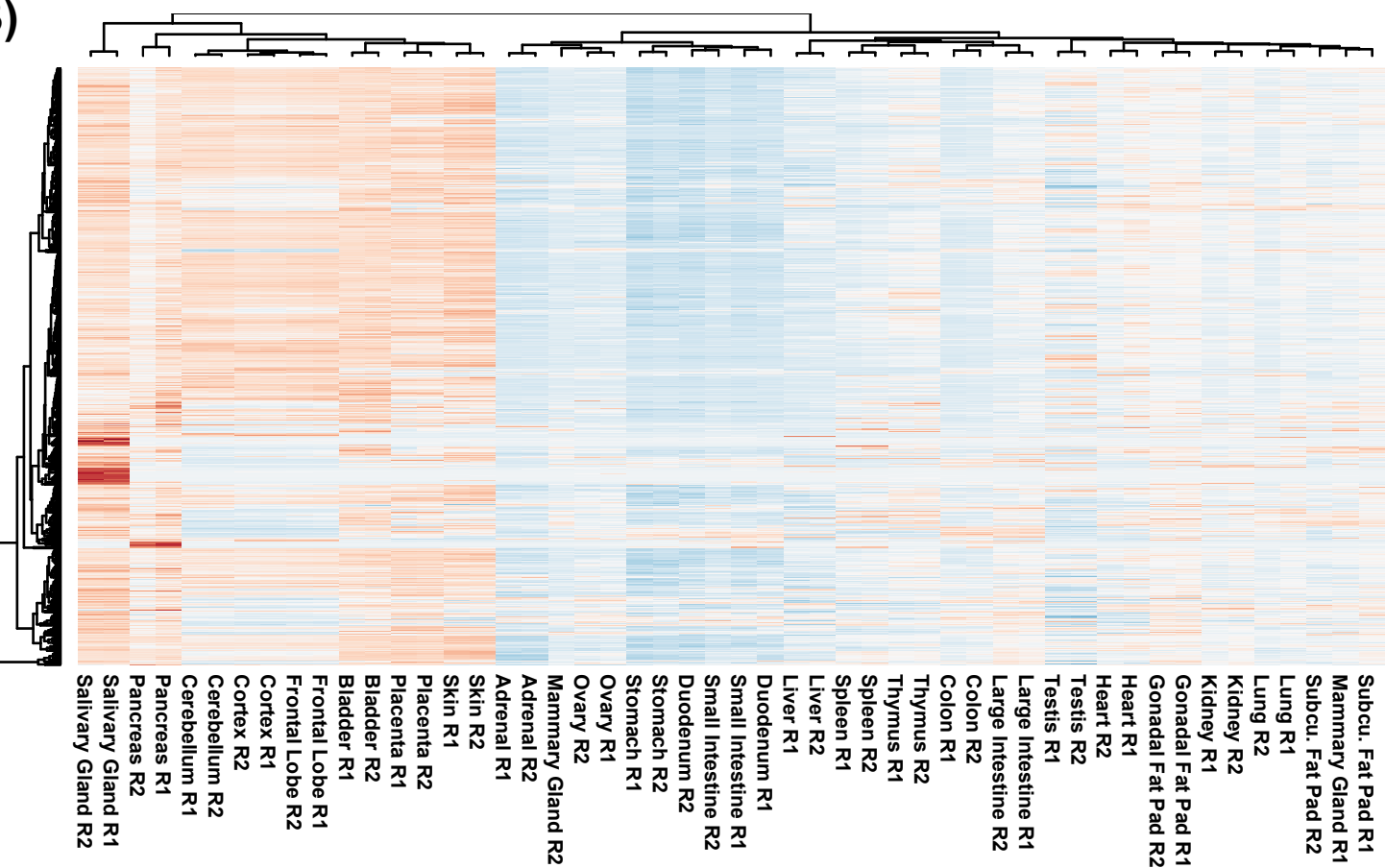

Supplement: Additional file 3: Figure S3. — Hierarchal clustering of the common genes expressed in salivary gland, pancreas, skin, bladder and placenta. A) Clustering of genes that collectively show higher expression in the salivary gland, pancreas, skin, bladder and placenta in comparison to all other tissues. B) Clustering of highly expressed genes shared between the two exocrine glands, the salivary gland and pancreas, in comparison to all other tissues. (PDF 11034 kb) [file 12864_2016_3228_MOESM3_ESM.pdf]

**Ascl3**

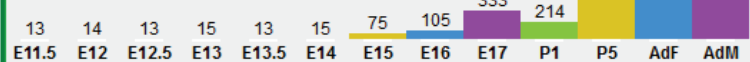

**Six1**

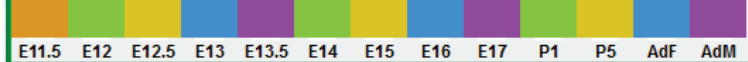

**Elf5**

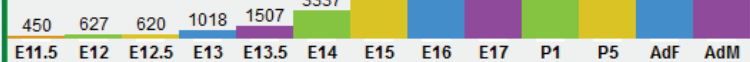

**Ehf**

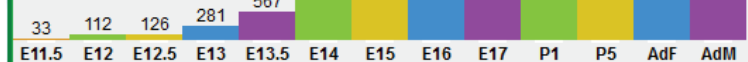

**Eaf2**

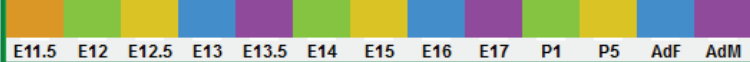

**Foxi2**

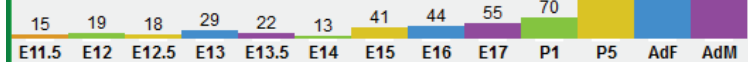

Supplement: Additional file 4: Figure S4. — Confirmation in expression of select transcription factors which comprise the adult salivary gland gene signature. Probe intensity levels of a subset of transcriptions factors which make up the salivary gland gene signature, as provided through the Salivary Gland Molecular Anatomy Project, are shown. Various stages of development and adult are included. P1- postnatal day 1, P5- postnatal day 5, AdF – adult female, AdM- adult male. (PDF 1355 kb) [file 12864_2016_3228_MOESM4_ESM.pdf]

Human Protein Atlas

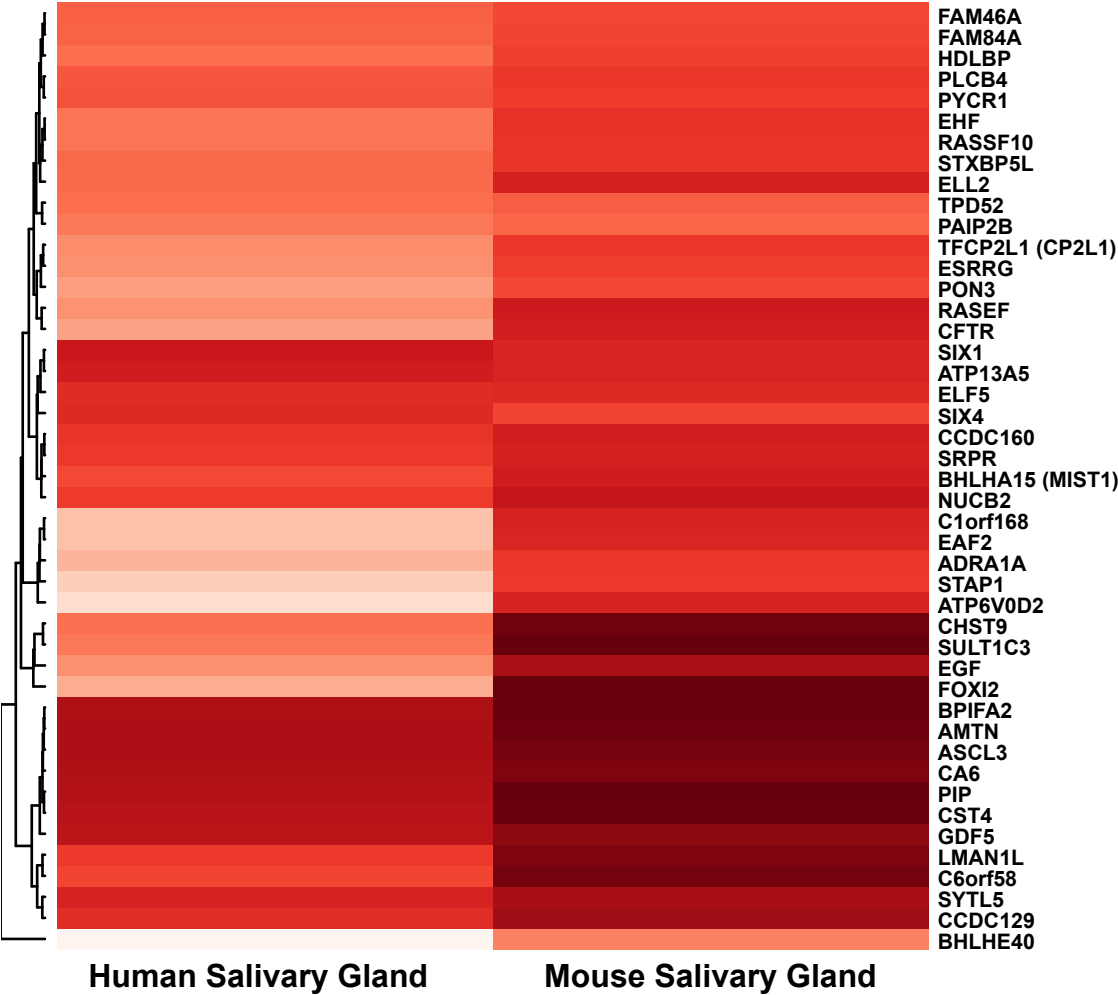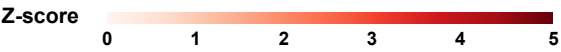

Supplement: Additional file 5: Figure S5. — Heatmap depicting the hierarchical clustering of the 45 genes that are conserved between the mouse adult salivary gland gene signature and the RNA-seq data obtained from the Human Protein Atlas. The values reported represent Z-scores of the conserved genes in their respective datasets. (PDF 737 kb) [file 12864_2016_3228_MOESM5_ESM.pdf]

CAGE Tissue Transcriptome

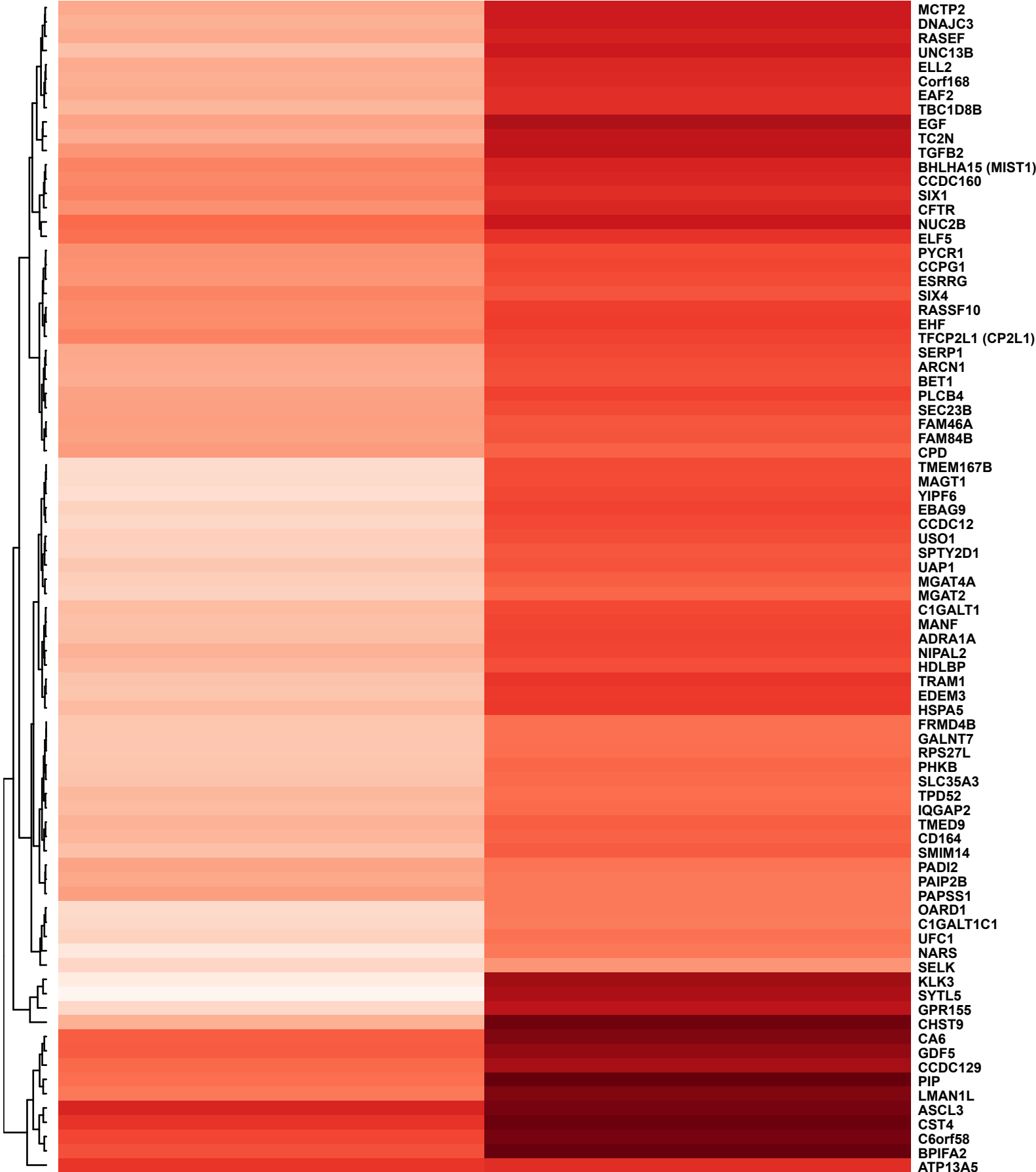

Human Submandibular Gland

Mouse Salivary Gland

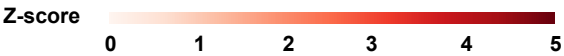

Supplement: Additional file 6: Figure S6. — Hierarchical clustering of the 82 genes that are conserved between the mouse adult salivary gland gene signature and the Cap Analysis Gene Expression (CAGE) datasets. The human submandibular gland is shown as a comparison. The values visualized in this heatmap represent standardized expression levels of the selected conserved genes relative to their respective datasets. (PDF 751 kb) [file 12864_2016_3228_MOESM6_ESM.pdf]
